# Comparative Study of Biological Characteristics, and Osteoblast Differentiation of Mesenchymal Stem Cell Established from *Camelus dromedarius* Skeletal Muscle, Dermal Skin, and Adipose Tissues

**DOI:** 10.3390/ani11041017

**Published:** 2021-04-04

**Authors:** Young-Bum Son, Yeon Ik Jeong, Yeon Woo Jeong, Mohammad Shamim Hossein, Alex Tinson, Kuhad Kuldip Singh, Woo Suk Hwang

**Affiliations:** 1 UAE Biotech Research Center, Abu Dhabi 30310, United Arab Emirates; ybs@adbrf.org (Y.-B.S.); youniks@adbrf.org (Y.I.J.); doctorj1@adbrf.org (Y.W.J.); shamim0976@gmail.com (M.S.H.); 2 Hilli E.T. Cloning and Surgical Centre Presidential Camels and Camel Racing Affairs, Al-Ain P.O. Box 17292, Unitied Arab Emirates; heffoundation@hotmail.com (A.T.); kskuhad@hotmail.com (K.K.S.)

**Keywords:** mesenchymal stem cells, *Camelus dromedarius*, skeletal muscle, dermal skin, adipose tissue, differentiation

## Abstract

**Simple Summary:**

Mesenchymal stem cells (MSCs) can be isolated in various types of tissues and exhibit different characteristics. In this study, MSCs were established from skeletal muscle, dermal skin, and adipose tissue from a single *Camelus dromedarius* donor. We also identified an efficient source for osteoblast differentiation and analyzed biological characteristics.

**Abstract:**

Mesenchymal stem cells (MSCs) showed in vitro mesoderm-lineage differentiation and self-renewal capacity. However, no comparative study was reported on the biological characteristics of stem cells derived from skeletal muscle (SM-MSCs), dermal skin (DS-MSCs), and adipose tissues (A-MSCs) from a single donor in camels. The present study aimed to evaluate the influence of MSCs source on stem cell characteristics. We evaluated proliferation capacity and mesoderm-lineage differentiation potential from SM-MSCs, DS-MSCs, and A-MSCs. They showed spindle-like morphology after homogenization. The proliferation ability was not significantly difference in any of the groups. Furthermore, the portion of the cell cycle and expression of pluripotent markers (Oct4, Sox2, and Nanog) were similar in all cell lines at passage 3. The differentiation capacity of A-MSCs into adipocytes was significantly higher than that of SM-MSCs and DS-MSCs. However, the osteoblast differentiation capacity of A-MSCs was significantly lower than that of SM-MSCs and DS-MSCs. Additionally, after osteoblast differentiation, the alkaline phosphatase (ALP) activity and calcium content significantly decreased in A-MSCs compared to SM-MSCs and DS-MSCs. To the best of our knowledge, we primarily established MSCs from the single camel and demonstrated their comparative characteristics, including expression of pluripotent factors and proliferation, and in vitro differentiation capacity into adipocytes and osteoblasts.

## 1. Introduction

The *Camelus dromedarius* is a domesticated camel and is a unique animal providing milk and meat in desert areas because of its physiological adaptation to high temperature, intense sunlight, and adaptability to water shortages [1,2]. Furthermore, camel racing is a very popular sport in the gulf areas and has great economic value [2]. As these racing camels are trained from a young age, bone diseases, including bone fractures, are becoming a problem, which is pointed out as a major cause of lameness in animals [3]. Generally, minor bone defects due to fractures frequently appear, and most of them are regenerated into original tissues, but extensive bone defects due to severe trauma are difficult to treat [4]. Autogenous bone graft has been attempted as a treatment for these extensive bone defects [5]. However, collecting autogenous bones accompanied by additional invasive surgery increases the risk of infection and pain [6]. Furthermore, the quantity of bones required for the grafts may be difficult to harvest and the quality of bone is also unpredictable [6,7]. Accordingly, attention has been focused on research for overcoming these bone diseases using stem cells.

Mesenchymal stem cells (MSCs) are a promising source of multipotent cells having self-replication, and multi-lineage capacity, including adipocytes, chondrocytes, and osteoblasts [8,9,10,11]. They are suitable for stem cell therapeutic applications without any ethical issues and immune-reaction [10,11]. Accordingly, several studies were reported on cell-based bone tissue engineering using MSCs in various mammalians [12,13,14]. Studies have found stem cells were transplanted into bond defects resulted in fusion [12]. Canine allogenic bone marrow-derived MSCs (BM-MSCs) transplanted into the site of critical-sized segmental bone defect, and the defect site was filled with new bone [13]. Furthermore, interesting studies confirmed that when human stem cells were transplanted with a scaffold into a miniature pig, the formation of new bones was confirmed at the transplant site, and it was reported that no immune rejection reaction was observed even when the xenogeneic stem cells were transplanted [9,14].

Applying an efficient cell source is essential for bone regeneration [15]. Therefore, there is increasing interest in improving the efficiency of differentiation into osteoblasts for bone regeneration. Several studies were reported on the isolation and culture of MSCs using various tissues including bone barrow [16,17,18,19]. However, BM-MSCs have lower proliferation and differentiation capacity than other sources of stem cells and show a small number of cells and donor-age-dependent characteristics [20]. Recently, many studies have been conducted on the source of MSCs that can replace bone marrow, and it has been reported that mesenchymal stem cells can be obtained from many tissues, including skeletal muscle, dermal skin, and adipose tissue [17,18,19]. Additionally, MSCs from skeletal muscle, dermal skin, and adipose tissue were reported for the presence of osteoblast differentiation capacity [21,22,23]. However, there was no comparative study of different sources of single donor-derived MSCs for the osteoblast differentiation in camel.

In the present study, the stem cells derived from a single donor of skeletal muscle (SM-MSCs), dermal skin (DS-MSCs), and adipose tissues (A-MSCs) were successfully established. We also evaluated osteoblast differentiation capacity on the basis of evaluation under the same induction conditions.

## 2. Materials and Methods

### 2.1. Chemicals and Media

We purchased all chemicals from Sigma (St. Louis, MO, USA) unless otherwise noted. 

### 2.2. Establishment and Culture of Skeletal Muscle, Dermal Skin, and Adipose Tissue-Derived Mesenchymal Stem Cells

The isolation and culture of mesenchymal stem cells from skeletal muscle (SM-MSCs), dermal skin (DS-MSCs), and adipose tissues (A-MSCs) were conducted following previously reported techniques with minor modification [8,10]. All animal procedures were conducted following the animal study guidelines, which were approved by the Institutional Animal Care and Use Committee (IACUC) at the Management of Scientific Centers and Presidential Camels (Accession No: PC4.1.5). In the present study, a total of four female camels aged from 4 to 5 years and weighing approximately 400 kg were used. The animals were sedated with ketamine hydrochloride (Ilium, Hlendenning, Australia; 0.25 mg/kg body weight) and xylazine (Ceva, Libourne, France; 0.25 mg/kg body weight) through intravenous injection using an 18-gauge needle. A meniscus pattern of 2 cm × 1 cm was infiltrated with local anesthetic on the left flank of the abdomen in front of the anterior crest of the ilium using 2% Lidocaine (Jeil, Daegu, Korea). The skin, fat, and muscle were exposed and obtained through a longitudinal incision in a meniscus shape (1.5 cm; depth and 2 cm × 1 cm diameter) using a combination of the scalpel blade, forceps, and surgical scissors. The incision site was covered with two layers using 2-0 vicryl and 2-0 nylon sutures. The obtained tissues washed two times in Dulbecco’s Phosphate-buffered saline (DPBS; Welgene, Gyeongsan, Korea) supplemented with 1% antibiotic-antimycotic (Thermo fisher scientific, Waltham, MA, USA). We minced the samples into small pieces with a surgical blade. They were dissociated with in high-glucose Dulbecco’s modified Eagle’s medium (DMEM; Thermo fisher scientific, Waltham, MA, USA) containing 0.1% collagenase type I (Thermo fisher scientific, Waltham, MA, USA) at 38 °C in a humidified atmosphere of 5% CO_2_ and O_2_ for 4 h. The cells released with collagenase were washed with high-glucose DMEM containing 10% fetal bovine serum (FBS; Invitrogen, Carlsbad, California, USA) by centrifugation at 300× *g* for 3 min and filtered through 40 µm nylon strainer (Falcon, Franklin, NJ, USA). After that, we cultured these cells in 60 mm culture dishes with high-glucose DMEM supplemented with 10% FBS, 1% nonessential amino acid (Thermo fisher scientific, Waltham, MA, USA), 1% antibiotic-antimycotic, and 0.1% β-mercaptoethanol (Thermo fisher scientific, Waltham, MA, USA) at 38 °C in a humidified incubator with 5% CO_2_ and O_2_. The culture media was changed every 48 h until confluence was reached 80%, and they were sub-cultured with 0.25% trypsin EDTA solution (Gibco, Paisely, UK) and cryopreserved in high-glucose DMEM containing 20% FBS and 10% dimethyl sulfoxide (DMSO).

### 2.3. Cell Proliferation Analysis

We conduct a population doubling time (PDT) assay to evaluate the cell proliferation capacity [8]. All cells were seeded into six-well plates (Nunc, NY, USA). Cells were counted using a hematocytometer for every passage at 72-h intervals. We calculated the PDT of the MSCs following the formula PDT = log2 × T/(logNC—logNI), where T is the cell culture time, logNC is the cultured cell number, and logNI is the initial cell number.

### 2.4. Cell Cycle Analysis

The culture SM-MSCs, DS-MSCs, and A-MSCs were fixed with 70% ethanol for 1 h at passage 3. The fixed cells were washed twice with DPBS and mixed with propidium iodide solution (10 μg/mL) and RNase A (100 μg/mL) for 15 min. The stained cells were analyzed by flow cytometry (BD FACSVerseTM, BD Biosciences, Franklin Lakes, New Jersey, USA) and categorized into G0/G1, S, and G2/M phases.

### 2.5. In Vitro Differentiation into Adipocyte, and Chondrocyte

To confirm the mesoderm-lineage differentiation, the SM-MSCs, DS-MSCs, and A-MSCs were differentiated at passage three into adipocyte, and chondrocytes as previously described with minor modifications [24]. Briefly, the cells were cultured with adipogenic-specific induction media for 21 days. The adipogenic induction medium consisted of DMEM supplemented with 10% FBS, 100 µM indomethacin, 10 µM insulin, and 1 µM dexamethasone. Adipogenic differentiation was confirmed by staining lipid droplets using oil red O staining. The chondrocyte differentiation was performed using conventional pellet culture techniques. In brief, 1 × 10^6^ cells at passage three were suspended with 1 mL of STEMPRO chondrogenesis differentiation media with 10% supplement in a 15 mL tube. The cell pellets were cultured for 3 weeks. To confirm the deposition of proteoglycans, the cell pellets were embedded paraffin section after dehydration. They were stained with 1% Alcian blue solubilized in 3% acetic acid for 10 min, with counterstaining of 0.1% nuclear fast red solution for 1 min.

### 2.6. In Vitro Differentiation into Osteoblast

The osteoblast differentiation was induced as previously reported with minor modifications [24]. All types of MSCs were cultured in DMEM containing 10% FBS, 10 nM dexamethasone, 50 μg/mL ascorbic acid, and 10 mM sodium ß-glycerophosphate for three weeks. The osteoblast induction media was changed every 48 h. Differentiated osteoblasts were fixed with 4% paraformaldehyde and stained with silver nitrate solution (Von kossa staining) and alizarin red S to confirm the mineralization and calcium depositions.

### 2.7. Real-Time Quantitative Polymerase Chain Reaction (RT-qPCR) Analysis

The expression of pluripotent markers and lineage-related genes was analyzed using real-time quantitative polymerase chain reaction (RT-qPCR). Total RNA was extracted using an easy-spin Total RNA Extraction Kit (Intron, Seongnam, Korea) and quantified using a Nanodrop 1000 spectrophotometer (Thermo Fisher Scientific, Waltham, MA, USA). To synthesize complementary DNA (cDNA), reverse transcription was conducted from total purified RNA (2 μg) using HisenScript RT PreMix kit (Intron, Seongnam, Korea) with 10 μM OligodT primer at 42 °C for 50 min. The RT-qPCR was conducted using a Rotor-Gene Q cycler (Qiagen, Hilden, Germany) and RealMODTM Green AP 5 × qPCR mix (Intron, Seongnam, Korea) containing 200 nM of forward and reverse primers. The amplification setting included denaturation at 95 °C for 60 s followed by 50 cycles of 95 °C for 10 min, 60 °C for 6 s, and 72 °C for 4 s. Gene expression was normalized to the mRNA levels of a reference gene, glyceraldehyde-3-phosphate dehydrogenase (GAPDH). The information of primers used in this study is listed in Table 1.

### 2.8. Alkaline Phosphatase Activity

Alkaline phosphatase (ALP) activity was assessed using a Tartrate-resistant acid phosphatase (TRACP) and ALP assay kit (Takara, Noji higashi, Japan) according to the manufacturer’s instructions. On day seven of osteoblast differentiation, cells were washed with DPBS and treated with 50 µL of extraction solution and substrate solution. The reaction was stopped using 50 µL of stop solution (0.5 N NaOH), and absorbance was evaluated using 405 nm by the VERSAmax microplate reader (Molecular Devices, San Jose, CA, USA). The cellular proteins were determined using the BCA protein assay kit (Thermo Fisher Scientific, Waltham, MA, USA). We calculated ALP activity values as IU/mg to the total cellular protein.

### 2.9. Calcium Colorimetric Assay

We measured calcium deposition using a calcium colorimetric assay kit (Biovision, Milpitas, San Francisco, CA, USA) according to the manufacturer’s instructions. The induced osteoblasts were treated with 0.6 N HCl for 24 h at 20 °C. After that, the supernatant was moved to each well of 96-well plates and mixed with 60 µL of calcium assay buffer and 90 µL of chromogenic reagent at 20 °C for 10 min. Absorbance at 575 nm was determined using the VERSAmax microplate reader (Molecular Devices, San Jose, CA, USA). We measured cellular proteins using the BCA protein assay kit (Thermo Fisher Scientific, Waltham, MA, USA). Calcium values were calculated as µg/mg to the total cellular protein.

### 2.10. Statistical Analysis

All data were analyzed by one-way analysis of variance (ANOVA) using SPSS version 23 (IBM), and Tukey’s test was conducted for between-group comparisons. Data were represented as mean ± standard deviation (SD), and *p* < 0.05 was considered significant.

## 3. Results

### 3.1. Establishment of MSCs Derived from Skeletal Muscle, Dermal Skin, and Adipose Tissues

**Figure 1:**
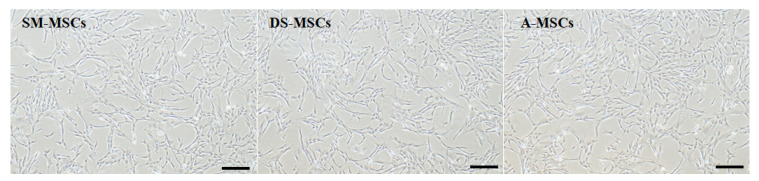
Cellular morphology of mesenchymal stem cells (MSCs) derived from skeletal muscle (SM-MSCs), dermal skin (DS-MSCs), and adipose tissue (A-MSCs) was observed at passage three by phase-contrast microscope. Scale bar = 100 µm.

**Figure 2:**
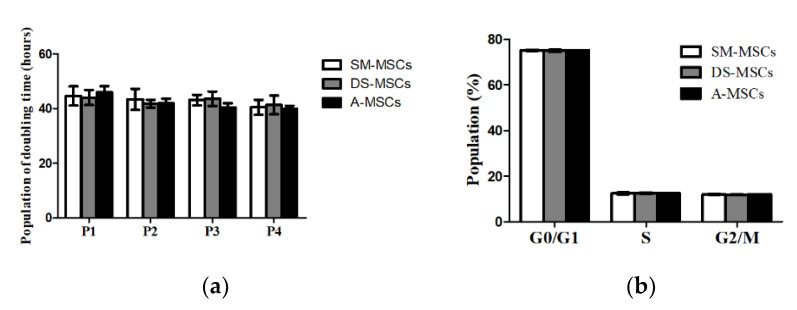
Cellular proliferation and cell cycle analysis of skeletal muscle (SM-MSCs), dermal skin (DS-MSCs), and adipose tissue (A-MSCs). (**a**). Population doubling time (PDT) analysis was performed in SM-MSCs, DS-MSCs, and A-MSCs. There was no significant difference during the passage. (**b**). The portion of the cell cycle was analyzed in SM-MSCs, DS-MSCs, and A-MSCs. The cell cycle arrest and DNA replication were similar in all three types of MSCs. The values were expressed as mean ± SD of four independent experiments.

**Figure 3:**
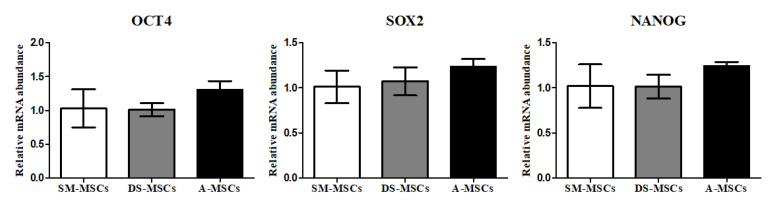
The expression levels of pluripotent markers of MSCs are derived from skeletal muscle (SM-MSCs), dermal skin (DS-MSCs), and adipose tissue (A-MSCs). There was no significant difference among all three types of MSCs. Data are represented by the mean ± SD of four independent experiments.

MSCs derived from skeletal muscle (SM-MSCs), dermal skin (DS-MSCs), and adipose tissue (A-MSCs) from a single donor were isolated and cultured. All the cells showed a spindle-like morphology and were confirmed homogenous adherent morphology after passage three (Figure 1). The proliferation capacity of cells was evaluated in passages one, two, three, and four. There was no difference in population doubling time (PDT) among the cells (Figure 2a). To assess the cell viability, we also analyzed cell cycle characteristics in all cells at passage three. Our data showed that the portion of the G0/G1 phase, and S phase, was the same in all groups (Figure 2b). All SM-MSCs, DS-MSCs, and A-MSCs expressed pluripotent factors (OCT4, SOX2, and NANOG). There was no difference in the expression of mRNA levels among the cells (Figure 3).

### 3.2. In Vitro Adipogenic and Chondrogenic Lineage Differentiation Capacity of MSCs

**Figure 4:**
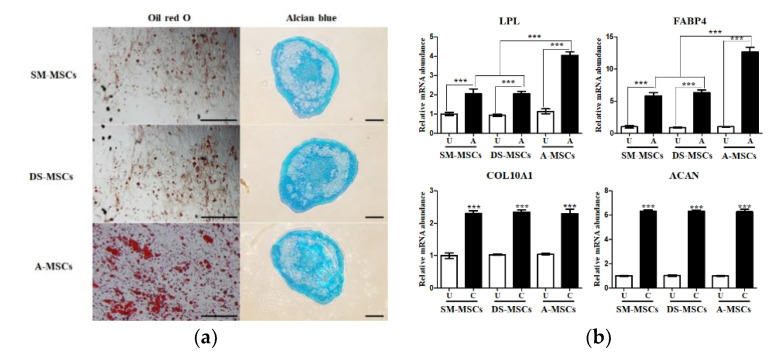
In vitro mesoderm-lineage (adipocyte and chondrocyte) differentiation of MSCs from three types of tissues. (**a**). Adipocytes and chondrocytes differentiation were confirmed oil red O (for adipocytes) and Alcian blue (for chondrocytes) staining. (**b**). The gene expression of adipocytes and chondrocytes-specific markers in differentiated cells. Data are represented by the mean ± SD of four independent experiments. Asterisks indicate significant difference between the undifferentiated cells and differentiated cells (*** *p* < 0.001). White bar: undifferentiated cells; black bar: differentiated cells; U: undifferentiated cells; A: adipogenic differentiated cells; C: chondrogenic differentiated cells.

The mesoderm-lineage (adipocyte and chondrocyte) differentiation potential of SM-MSCs, DS-MSCs, and A-MSCs was evaluated. Cytochemical staining confirmed that cells induced into adipocyte and chondrocyte, as verified by the intracellular lipid droplets and accumulation of proteoglycan by oil red O, and Alcian blue staining, respectively (Figure 4a).

The adipocyte- and chondrocyte-specific genes were analyzed before and after the differentiation by RT-qPCR (Figure 4b). The adipocyte-specific genes, i.e., lipoprotein lipase (LPL) and fatty acid-binding protein 4 (FABP4), showed significantly (*p* < 0.001) higher expression in differentiated adipocytes compared to the non-differentiated cells. Furthermore, our data showed that the expression of LPL and FABP4 was significantly increased in the differentiated cells derived from A-MSCs compared to those derived from SM-MSCs, and DS-MSCs. The expression of type X collagen gene (COL10A1) and agreecan (ACAN), chondrogenesis markers, in all the three types of MSCs significantly (*p* < 0.001) increased after differentiation. However, no significant difference in the expression of COL10A1 and ACAN was confirmed in the differentiated chondrocytes from the three groups (Figure 4b).

### 3.3. In Vitro Osteogenic Differentiation Capacity of MSCs

**Figure 5:**
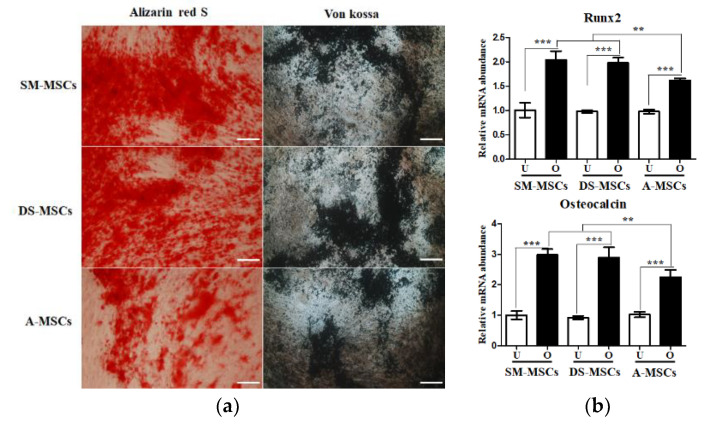
The results of in vitro osteoblast differentiation of MSCs derived from skeletal muscle (SM-MSCs), dermal skin (DS-MSCs), and adipose tissue (A-MSCs). (**a**). Cytochemical staining of differentiated osteoblasts. Scale bar = 200 µm. (**b**). Results of RT-qPCR for osteoblast-specific markers in differentiated cells. All osteoblast-specific markers were significantly higher in differentiated osteoblast from SM-MSCs and DS-MSCs than that from A-MSCs. Data are represented by the mean ± SD of four independent experiments. Asterisks indicate significant difference between the undifferentiated cells and differentiated cells (** *p* < 0.01; *** *p* < 0.001). White bar: undifferentiated cells; black bar: differentiated cells; U: undifferentiated cells; O: osteogenic differentiated cells.

**Figure 6:**
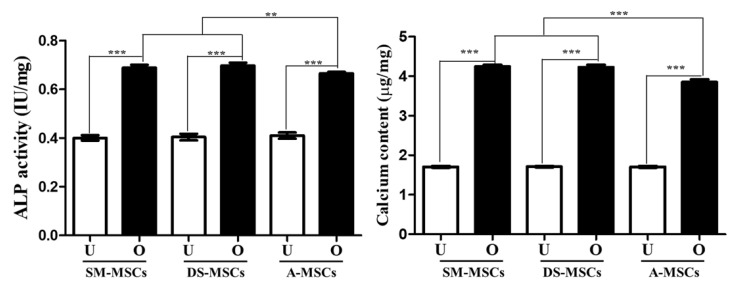
The levels of alkaline phosphatase (ALP) activity and calcium content of MSCs derived from skeletal muscle (SM-MSCs), dermal skin (DS-MSCs), and adipose tissue (A-MSCs). The differentiated osteoblasts from SM-MSCs and DS-MSCs showed significantly (*p* < 0.05) increased the ALP activity (day 7) and calcium content (day 21) compared to that from A-MSCs. Data are represented by the mean ± SD of four independent experiments. Asterisks indicate significant difference between the undifferentiated cells and differentiated cells (** *p* < 0.01; *** *p* < 0.001). White bar: undifferentiated cells; black bar: differentiated cells; U: undifferentiated cells; O: osteogenic differentiated cells.

The SM-MSCs, DM-MSCs, and A-MSCs were successfully differentiated into osteoblasts. The calcified extracellular matrix formation was confirmed by Alizarin red S, and Von kossa staining (Figure 5a). The expression of osteoblast-specific markers (Runx2, and Osteocalcin) was significantly higher in the osteogenic-induced MSCs than the non-differentiated cells (Figure 5b). Additionally, upon the induction of osteogenesis, the expression of Runx2 and Osteocalcin was significantly (*p* < 0.01) higher in SM-MSCs and DS-MSCs, compared to A-MSCs. We also assessed ALP activity and calcium content. The ALP activity significantly (*p* < 0.01) increased differentiated osteoblasts derived from SM-MSCs and DS-MSCs, compared to those derived from A-MSCs at seven days after osteogenic-differentiation (Figure 6). Furthermore, we also confirmed that calcium content was also significantly (*p* < 0.001) increased in SM-MSCS and DS-MSCs 21 days after differentiation into osteoblast (Figure 6).

## 4. Discussion

The bone is a hard calcified organ that consists of proteins such as collagen and hydroxyapatite [25]. Unlike other tissues, bones undergo mineralization process and remodeling process [25,26]. Osteoblasts play important roles in producing proteins that form bone and accumulating minerals [27]. Bone-related problems, including bone fracture and bond defect, can become fatal unless properly treated. Traditionally, these disorders can be treated by using biological bone grafts [28]. Although bone grafts can yield satisfactory results, clinical use is limited due to the lack of available bone, additional invasive surgery, and risk of infections. Therefore, stem cell-based therapy has emerged as an alternative method for bone regeneration.

As described above, bone marrow-derived MSCs (BM-MSCs) were the first known stem cells, but stemness and multi-potency differentiation capacity are limited depending on donor aging [20]. Furthermore, until now, studies on biological characteristics, including the establishment of stem cells, and osteoblast differentiation from various tissues of camels, has been limited. The present study was performed to evaluate the stemness and multi-potency of MSCs derived from different camel tissues. We successfully isolated and cultured SM-MSCs, DS-MSCs, and A-MSCs from a single donor to confirm the most efficient source for osteoblast differentiation. We compared the morphology, proliferation, expression of pluripotent markers, and in vitro differentiation into mesoderm-lineage including osteoblasts in SM-MSCs, DS-MSCs, and A-MSCs. All types of MSCs showed spindle-like morphology under identical conditions. The pluripotent factors play an important role in the proliferation and differentiation of MSCs [29,30]. In this study, MSCs from skeletal muscle, dermal skin, and adipose tissues expressed critical pluripotent markers such as OCT4, SOX2, and NANOG at mRNA levels. Our data also showed that there was no difference in proliferation rate and expression of pluripotent markers among the cells. The expression of pluripotent markers revealed the stemness capacity of MSCs derived from skeletal muscles, dermal skin, and adipose tissues.

We demonstrated that SM-MSCs, DS-MSCs, and A-MSCs were successfully differentiated into adipocytes under specific culture conditions (Figure 4a). The gene expression of adipogenic markers, LPL, and FABP4 were significantly increased in differentiated adipocytes compared to the non-differentiated cells. Additionally, adipose tissue-derived MSCs revealed higher differentiation potential into adipocytes compared to skeletal muscle, and dermal skin-derived MSCs. This finding is similar to previous studies [31,32]. Stem cells derived from certain tissue show elevated differentiation efficiency depending on the environment where tissues are located [31]. Similarly, all MSCs differentiated into chondrocytes and expressed chondrocyte-related markers, i.e., the type X collagen gene (COL10A1) and agreecan (ACAN), which are following the previous study [31].

The present study also assessed the differentiation potential of SM-MSCs, DS-MSCs, and A-MSCs toward osteoblasts. Several studies demonstrated the capacity of various camel stem cells differentiated toward osteoblasts [2,33,34]. However, the comparative evaluation of osteoblast differentiation ability of different regions to derive MSCs in camels from an osteogenic lineage has not been reported. In the process of osteoblast differentiation, it is reported that the expression of collagen type I and Runx2 increases in the initial proliferative phase, and calcium accumulates with an increased in the expression of ALP and osteocalcin (OCN) during the formation of mineral matrix formation [28,35]. We also confirmed that after osteoblast differentiation, the osteoblast-specific genes (Runx2, and Osteocalcin) were significantly higher in differentiated cells compared to undifferentiated cells. Furthermore, the differentiated osteoblasts from SM-MSCs and DS-MSCs showed significantly higher expression of osteoblast-specific markers than that from A-MSCs.

ALP activity, mineral matrix formation, and calcium deposition are crucial markers on osteoblast [35,36,37]. The transporting of calcium ions in osteoblast-induced mineralization and calcium deposition meant mineralization process in osteoblast differentiation [38,39]. Our data showed that the expression of ALP was significantly increased in differentiated osteoblast compared to non-differentiated cells. Interestingly, osteoblasts from SM-MSCs and DS-MSCs exhibited significantly higher expression of ALP activity than that from A-MSCs. Similarly, the levels of calcium content were also lowest in induced A-MSCs groups. Although additional studies will be necessary to evaluate the underlying mechanisms, the present study suggests that MSCs from different tissues have varied differentiation capacity into different lineage even if their identical donor origin.

## 5. Conclusions

MSCs established from a single donor of skeletal muscle, dermal skin, and adipose tissues showed similar characteristics related to cellular morphology, proliferation, expression of pluripotent marker, and differentiation into chondrocytes. All types of MSCs successfully differentiated toward adipocyte and osteoblasts. However, the expression of adipocyte-specific markers revealed that adipose tissue-derived MSCs are notable sources for differentiation toward adipocytes. Furthermore, the expression of osteoblast-specific markers, ALP activity, and calcium content revealed that SM-MSCs and DS-MSCs are prominent sources for stem cell-based therapy. These in vitro results provide for the potential treatment of *Camelus dromedarius* utilizing MSCs with excellent osteoblast differentiation capacity. MSCs pre-establishment can allow for direct treatment following bone-related problems, including bone defects and fractures due to severe trauma. This approach is warranted to promote animal welfare by providing immediate relief to injuries, ameliorating suffering time, and reducing the risk for secondary injuries. To our knowledge, this is the first study in which the osteoblast differentiation capacity of MSCs from three different tissues from a single donor has been compared and a prominent MSCs source for the generation of osteoblasts has been demonstrated.

## Figures and Tables

**Table 1:** Lists of camel primers used in real-time quantitative polymerase chain reaction (RT-qPCR) analysis.

Gene Name (Symbol)	Primers Sequence	Product Size (bp)	Anneal. Temp (°C)
POU class 5 homeobox 1 (OCT4)	F: CGAGAGGATTTTGAGGCTGC R: GAGTACAGTGTGGTGAAGTGAG	122	60
Sex determining region Y-box 2 (SOX2)	F: CTCGCAGACCTACATGAACG R: TGGGAGGAAGAGGAAACCAC	144	60
Nanog homeobox (NANOG)	F: AGCACAGAGAAGCAGGAAGA R: CCACCGCTTACATTTCATTC	213	60
Lipoprotein lipase (LPL)	F: GAGAGTGTTACCTACACCAA R: GCCTTTACTCTGATCTTCTC	248	60
fatty acid-binding protein 4 (FABP4)	F: GTGACCATCAGTGTGAATG R: GCACCTCCTTCTAAAGTTAC	152	60
the type X collagen gene (COL10A1)	F: TATCCAGCTATAGGCAGTC R: TCGTAGGTGTACATTACAGG	194	60
Agreecan (ACAN)	F: TGTGGAGGGTGTTACTGAAC R: GACTGATGACCCTTCTACCC	154	60
Runt-related transcription factor 2 (Runx2)	F: GACAGAAGCTTGATGACTCT R: GTAATCTGACTCTGTCCTTG	166	60
Osteocalcin	F: AGTGAGATGGTGAAGAGACT R: TAGGTTGTGCCGTAGAAG	176	60
Glyceraldehyde 3-phosphate dehydrogenase (GAPDH)	F: GCTGAGTACGTTGTGGAGTC R: TCACGCCCATCACAAACATG	133	60

## Data Availability

Data access can be requested on demand with the corresponding author.
